# Ratiometric analysis using Raman spectroscopy as a powerful predictor of structural properties of fatty acids

**DOI:** 10.1098/rsos.181483

**Published:** 2018-12-12

**Authors:** Lauren E. Jamieson, Angela Li, Karen Faulds, Duncan Graham

**Affiliations:** Centre for Molecular Nanometrology, WestCHEM, Department of Pure and Applied Chemistry, Technology and Innovation Centre, University of Strathclyde, 99 George Street, Glasgow G1 1RD, UK

**Keywords:** Raman, fatty acids, lipids, ratiometric, food oils

## Abstract

Raman spectroscopy has been used extensively for the analysis of biological samples *in vitro*, *ex vivo* and *in vivo*. While important progress has been made towards using this analytical technique in clinical applications, there is a limit to how much chemically specific information can be extracted from a spectrum of a biological sample, which consists of multiple overlapping peaks from a large number of species in any particular sample. In an attempt to elucidate more specific information regarding individual biochemical species, as opposed to very broad assignments by species class, we propose a bottom-up approach beginning with a detailed analysis of pure biochemical components. Here, we demonstrate a simple ratiometric approach applied to fatty acids, a subsection of the lipid class, to allow the key structural features, in particular degree of saturation and chain length, to be predicted. This is proposed as a starting point for allowing more chemically and species-specific information to be elucidated from the highly multiplexed spectrum of multiple overlapping signals found in a real biological sample. The power of simple ratiometric analysis is also demonstrated by comparing the prediction of degree of unsaturation in food oil samples using ratiometric and multivariate analysis techniques which could be used for food oil authentication.

## 1. Introduction

Raman spectroscopy is recognized as a powerful tool for analysing biological samples due to its label-free and non-destructive nature, along with the abundance of chemically specific information it provides [1]. It has been demonstrated for *in vitro*, [2] *ex vivo*, [3] and *in vivo* [4] analysis, including being used in the clinic for intraoperative guidance during brain tumour resection [5,6]. A single Raman spectrum acquired from a biological sample contains a massive volume of information, consisting of multiple overlapping peaks from all Raman-active components in the biosample, e.g. proteins, nucleic acids and lipids. To understand the significance of a result, it is necessary to understand the origin of the underlying signals in these spectra, but due to the overwhelming number of molecules contributing to a single spectrum, this is not an easy task. While some assignments are essentially unambiguous, for example, the strong characteristic phenylalanine band at *ca* 1004 cm^−1^, [7] it is common practice that most assignments are made only ‘tentatively’ or assigned very broadly to, for example, ‘protein’ or ‘lipid’. These can be based on logical and educated arguments, which could be aided by, and also biased by, prior knowledge of the biological sample being investigated and the expected biochemical changes therein. Often, assignments are made by searching literature for evidence of previous assignments of the same peaks, with use­­ful publications, such as that by Talari *et al.* [8], collating an extensive list of Raman assignments of biomolecules with references.

It is also common practice in biological Raman studies to use multivariate analysis to group samples and identify the major sources of spectral variation between them, with the authors then hypothesizing the source of these spectral differences from a biological context [9]. Again these hypotheses are often formed by assigning the identified sources of spectral variation ‘tentatively’ and broadly. While these multivariate techniques are very powerful, reducing data dimensionality in large datasets, they often involve extensive pre-processing of data and could overlook subtler but important changes in biological systems. Careful analysis of spectra prior to such techniques may allow more robust and informative assignments to be made.

To better understand and assign these subtle changes in Raman spectra of biological samples, it is important to simplify the system and consider the spectra and respective assignments of individual component parts. In an idealistic situation, this would involve acquiring spectra of pure components of all potential biomolecules contributing to a sample. Realistically, this is virtually impossible due to the overwhelmingly large number of species present in typical biological samples, such as cells and tissues. However, by identifying a handful of the major components contributing to a biological Raman spectrum, it is possible to build up a bank of very specific spectral assignments, and recognize and identify simple trends in, for example, peak position and intensity, that relate to major characteristic features within families of biomolecules.

Previous studies have attempted to measure and assign peaks for individual pure components that are likely to be present in complex biosamples [10–12]. Attempts have also been made to understand spectral components by ‘fitting’ a series of ‘pure’ spectra to the spectrum of the complex biological sample. For example, a study from Stone *et al.* [13] used least squares fitting to fit spectra of the pure components actin, collagen I, collagen III, choline, triolein, oleic acid, DNA and cholesterol to cancerous and non-cancerous bladder and prostate tissue spectra. While this begins to allow a more detailed understanding of these individual constituents, it only considers a few specific constituents. Another increasingly popular multivariate technique for resolving constituent components from a biological Raman spectrum is multivariate curve resolution; however, the extent to which this can resolve individual biochemical species as opposed to broader classes of species is still limited [14–16].

A comprehensive study by Czamara *et al.* [17] provides a detailed review of Raman spectral assignments for a number of common lipid species. Lipid species have a particularly high Raman cross-section due to their non-polar nature, with highly polarizable C–H and C–C bonds making up the majority of the molecules. In addition, there is a strong need for better analytical techniques for measuring lipid species. The gold standard biological imaging technique, fluorescence spectroscopy, is particularly poor for imaging lipids due to lack of inherent fluorescence, and bulky fluorescent tags that can interfere with native lipid distribution and dynamics [18]. The alternative is to use mass spectrometry; however, this is destructive and, depending on the ionization method, may not detect all lipid species in a sample [19]. The label-free and non-destructive technique of Raman spectroscopy is therefore a highly attractive tool for understanding lipids in biological systems; however, there is a need for Raman to increase its analytical scope by providing more detailed and species-specific information return, bringing it closer to the capabilities of mass spectrometry in this respect. This study aims to create a solid foundation for building such an analytical Raman-based approach.

Both Raman and infrared spectroscopy have been applied extensively for the characterization of lipid species over the last five decades. Many of the early studies focused on the analysis of membrane lipids, most significantly phosphatidylcholines and interactions and changes in these in response to external stimuli [20–25]. Previous investigations have also recognized some characteristic trends with spectral features, in particular correlated with degree of unsaturation—with Raman peaks at 3013 cm^−1^, 1663 cm^−1^ and 1264 cm^−1^ correlating strongly with fatty acid (FA) unsaturation [26,27]. There are, however, very few systematic studies that analyse collective groups of lipid species and identify progressive trends in Raman spectra correlated to stepwise structural changes. This work is focused on analysing progressive changes in Raman peak ratios with respect to key structural properties—namely chain length and degree of saturation—of an important class of lipid species—FAs. Beyond simple spectral assignments of lipid species, the importance of this study lies in the systematic approach taken in considering a carefully selected group of FA species and correlative changes in Raman peak ratios with key structural features of the species that represent progressive structural changes. The Raman peak ratios include and also go beyond those considered previously and provide a fully comprehensive and logical spectral analytic approach. The ultimate goal of this work is to allow structural information to be easily extracted from spectra of multiplex mixtures of FAs and eventually in complex biological samples to gain superior information return than is currently possible. Instead of putting high reliance on multivariate analysis methods, the simple ratiometric approach used in this work is employed to demonstrate the strength in the careful inspection and assignment of Raman spectra themselves to identify spectral changes informed by chemical and spectroscopic knowledge that show clear systematic trends with structural features of biomolecules—here FAs. It is hypothesized that where key Raman peak ratios successfully correlate with structural information, analysis of multiplexed spectra could be performed more accurately than multivariate techniques where the addition of multiple further data points to models could potentially mask and dampen the changes in the most strongly correlated peaks.

To demonstrate the strength of this approach, simple multiplexed samples that consist predominantly of a mixture of triacylglycerides, with wide global interest, are analysed to determine their composition in terms of average FA chain length and degree of unsaturation—food oils. Food adulteration is a widespread global concern, and food oil adulteration contributes significantly to this [28]. In particular, adulteration of olive oil with cheaper vegetable oils is common practice in food crime [29,30]. Analytical methods to detect and combat this are sought after and both Raman spectroscopy [31–33] and infrared spectroscopy [34] have been studied to this end as well as other techniques such as mass spectrometry [35]. Many of the currently available methods rely on multivariate analysis for the identification of adulterated samples. Our approach provides an alternative analysis pathway using simple ratios, which could rapidly identify adulterated food oils based on FA chain length and saturation.

We present this study as the first in a series of progressively more complex ratiometric-based analyses for characterization of multiplexed lipid mixtures, ultimately biological cells and tissue, where more species-specific information will be gained. This will build up an extensive and inclusive platform for interpretation of Raman spectra of complex biological samples, which aims to expand the capabilities of Raman spectroscopy to provide an information return more similar to techniques such as mass spectrometry, but with the non-destructive advantage of Raman analysis.

## 2. Material and methods

### 2.1. Lipid compounds

FAs were purchased from Sigma-Aldrich. Table 1 summarizes their properties. Vegetable oil, sunflower oil and olive oil were purchased from Tesco and coconut oil was purchased from Lidl.

**Table 1.** Name, abbreviations, structure and properties of lipid species selected for analysis in this work.

name	abbreviation	structure	chain length (no. C)	degree of unsaturation (no. C = C)	state	no. CH_3_	no. CH_2_	no. H−C=
myristic acid	MA	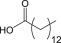	14	0	solid	1	12	0
palmitic acid	PA	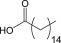	16	0	solid	1	14	0
stearic acid	SA	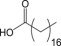	18	0	solid	1	16	0
arachidic acid	AA	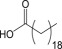	20	0	solid	1	18	0
behenic acid	BA	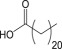	22	0	solid	1	20	0
palmitoleic acid	POA	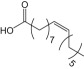	16	1 (9Z)	liquid	1	12	2
oleic acid	OA	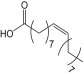	18	1 (9Z)	liquid	1	14	2
elaidic acid	EA	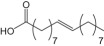	18	1 (9E)	solid	1	14	2
petroselinic acid	PSA	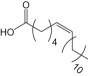	18	1 (6Z)	solid	1	14	2
linoleic acid	LA	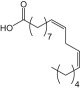	18	2 (9Z, 12Z)	liquid	1	12	4
α-linolenic acid	ALA	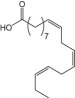	18	3 (9Z, 12Z, 15Z)	liquid	1	10	6
arachidonic acid	ADA	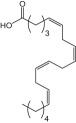	20	4 (6Z, 9Z, 12Z, 15Z)	liquid	1	10	8
stearidonic acid	SDA	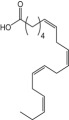	18	4 (5Z, 8Z, 11Z, 14Z)	liquid	1	8	8

### 2.2. Instrumentation

Raman spectra were acquired on a Renishaw inVia Raman microscope equipped with a 20×/NA 0.40 objective or a 50×/NA 0.75 objective and

(a) 532 nm Nd:YAG laser and 1800 l mm^−1^ grating giving a maximum power of 500 mW and a spectral resolution of *ca* 4.1 cm^−1^ measured using the full width at half maximum (FWHM) of the peak at 520 cm^−1^ in the spectrum of a silicon reference sample;
(b) 633 nm He-Ne laser and 1800 l mm^−1^ grating giving a maximum power of 500 mW and a spectral resolution of *ca* 3.5 cm^−1^ measured using the FWHM of the peak at 520 cm^−1^ in the spectrum of a silicon reference sample;
(c) 785 nm diode laser and 1200 l mm^−1^ grating giving a maximum power of 500 mW and a spectral resolution of *ca* 4.8 cm^−1^ measured using the FWHM of the peak at 520 cm^−1^ in the spectrum of a silicon reference sample.

### 2.3. FA spectral acquisitions

Spectra of each of the pure FAs were acquired after transferring a small quantity of the compound onto a CaF_2_ window. Spectra were acquired using a 20× objective, 10 s acquisition time and 50% laser power with spectra centred at both 1300 cm^−1^ and 3000 cm^−1^ and using 532 nm (20 mW laser power), 633 nm (10 mW laser power) and 785 nm (95 mW laser power) excitation. Three spectra were acquired for each FA at each spectral centre.

### 2.4. Food oil spectral acquisitions

Spectra of coconut oil, vegetable oil, sunflower oil and olive oil were acquired after transferring a small quantity of the oil onto a CaF_2_ window (coconut oil was melted first using warm tap water). Spectra were acquired using a 50× objective, 10 s acquisition time and 50%/20 mW laser power with spectra centred at both 1300 cm^−1^ and 3000 cm^−1^ and using 532 nm excitation (N.B. for vegetable oil at 3000 cm^−1^ an acquisition time of 1 s instead of 10 s was used due to high background fluorescence). Three spectra were recorded per sample. A different objective (50× instead of 20×) was used for food oil spectral acquisitions; however, this should not affect the resultant analysis, as instrument calibrations were always performed on a silicon standard prior to spectral acquisition to ensure consistent spectral response, and appropriate background corrections were performed on all spectra. The only notable difference observed in spectra between the two objectives was a higher overall spectral intensity when using the 50× objective due to a shorter working distance.

### 2.5. Data analysis

Spectra were processed in Matlab R2016a to smooth and subtract a baseline. For visualization, spectra were scaled between 0 and 1 and offset in increments of 1. Three spectra for each FA were recorded and the mean spectrum for each was plotted with shaded error bars showing standard deviation. For determination of ratios for saturated FAs, the intensities at 2850 cm^−1^ and 2935 cm^−1^ were extracted. In addition, the peak position of the peak at approximately 1100 cm^−1^ was recorded. For determination of ratios for unsaturated FAs, the intensities at 1262 cm^−1^, 1438 cm^−1^, 1655 cm^−1^, approximately 3005 cm^−1^ (as this peak shifted, the intensity for the peak maximum was taken in each case), 2850 cm^−1^ and 2935 cm^−1^ were extracted. In addition, the peak position of the peak at approximately 3005 cm^−1^ was recorded. These values were used to create the following plots where mean ± s.d. for each point was plotted:

— number of C versus peak position at approximately 1100 cm^−1^ for saturated FAs (position of this gauche C–C stretching peak is dependent on FA chain length)
— number of C versus 2850 cm^−1^ intensity/2935 cm^−1^ intensity for saturated FAs (intensity C–H (CH_2_) stretching/intensity C–H (CH_3_) stretching, expected to increase with increasing FA chain length for saturated FAs due to a corresponding increase in CH_2_/CH_3_ groups)
— number of C=C versus 1262 cm^−1^ intensity/1438 cm^−1^ intensity for unsaturated FAs (intensity =C−H deformation/intensity CH_2_ twisting, expected to increase with increasing number of C=C due to a higher number of =C–H and lower number of CH_2_ groups for the same chain length)
— number of C=C/number of CH_2_ (table 1) versus 1655 cm^−1^ intensity/1438 cm^−1^ intensity for unsaturated FAs (intensity C=C stretch/intensity CH_2_ twisting, expected to increase with increasing number of C=C due to a higher number of C=C and lower number of CH_2_ groups for the same chain length)
— number of C=C versus peak position at approximately 3005 cm^−1^ for unsaturated FAs (position of this =C−H stretch peak is dependent on saturation)
— number of H−C=/number of CH_2_ (table 1) versus approximately 3005 cm^−1^ intensity/2850 cm^−1^ intensity for unsaturated FAs (intensity =C–H stretch/intensity C–H (CH_2_) stretching, expected to increase with increasing number of C=C for the same chain length due to an increase in H–C= and decrease in CH_2_)
— number of CH_2_/number of CH_3_ (table 1) versus 2850 cm^−1^ intensity/2935 cm^−1^ intensity for unsaturated FAs (intensity C–H (CH_2_) stretch/intensity C–H (CH_3_) stretch, expected to reflect number of CH_2_/CH_3_ groups in FA chain).

Linear regression was performed on each plot using GraphPad Prism. The slope and *y*-intercept were recorded for each line for use in predictions for unknown spectra. In addition, GraphPad Prism was used to determine if the slopes of the best-fit lines determined by linear regression were significantly different for 532 nm, 633 nm and 785 nm laser excitation in each case.

Principal component analysis (PCA) and partial least squares regression (PLSR) were performed on spectra centred at 1300 cm^−1^ and spectra centred at 3000 cm^−1^ (cut to remove points higher than 3200 cm^−1^) in Matlab R2016a. Petroselinic acid only in liquid form was included and elaidic acid was omitted. In each case, all FA spectra (3 spectra for each FA) were combined into a single variable and scaled between 0 and 1. PCA was performed on both the low wavenumber and high wavenumber data in Matlab R2016a and a score plot of principal component 1 against principal component 2 was used to visualize the results. For PLSR, the data were randomly split into 70% training data and 30% test data. PLSR was performed in MATLAB R2016a on the training data using the MATLAB function plsregress and 10 components and a plot of estimated mean squared prediction error (MSPE) against number of components revealed a choice of 2 components to be optimal. PLSR was then performed on the training data using 2 principal components and the estimated MSPE and *R*^2^ for the training data were recorded. The resultant model was then applied to the test data and *R*^2^ determined and the results for both the training and test datasets were plotted as known against predicted number of C=C per FA.

For food oil samples, processing was again performed in Matlab R2016a using the same steps as were used for the pure FA spectra and spectra centred at 3000 cm^−1^ were cut to remove points higher than 3200 cm^−1^. For visualization, spectra were scaled between 0 and 1 and offset in increments of 1. Three spectra for each oil were recorded and the mean spectrum for each was plotted with shaded error bars showing standard deviation. For prediction of degree of unsaturation and chain length using the ratiometric method, the intensity and position of the peaks at each of the Raman shift values previously indicated were determined and used to predict these characteristics using the linear regression results previously determined. For prediction using PLSR, the model generated (using both low wavenumber and high wavenumber) was applied to the oil spectra to predict number of C=C per fatty acid.

## 3. Results and discussion

### 3.1. Fatty acid selection and data acquisition

To better understand and characterize spectral signatures of mixtures of unknown lipid species, a series of spectra for exemplary pure lipid species were acquired. For the purpose of this study, these were limited to free FAs in order to demonstrate the power of basic ratiometric data analysis for deconvolution of and understanding of spectral signatures in a multiplex mixture. Thirteen free FAs were selected, primarily based on their abundance in human biological samples, i.e. the most predominant FAs found in the body. In addition, some species were included to allow a systematic study on spectral detection of fundamental characteristics such as chain length and degree of unsaturation. Five saturated FAs were selected, ranging from C14 to C22; and eight unsaturated FAs were selected, ranging from a single C=C double bond to four C=C double bonds. All but one of these unsaturated FAs had all double bonds in the *cis*/Z configuration, as is predominantly found in nature, with the *trans*/E isomer of oleic acid, elaidic acid, being included to allow the difference between *cis* and *trans* isomers to be determined. Table 1 lists all selected compounds along with their structure, abbreviation that will be used for reference in this paper and some basic chemical characteristics of each species. To gain a better understanding of spectral features, it is important to return to fundamental chemistry, and thus a simple ratio of a peak intensity corresponding to a vibration associated with, for example, a CH_2_ group to a peak intensity of a vibration associated with a CH_3_ group should be reflected in the ratio of these groups in the molecule under investigation. Spectra of all species were acquired using 532 nm, 633 nm and 785 nm wavelength excitation. Spectra were acquired centred at 1300 cm^−1^ (low wavenumber (LWN)) and 3000 cm^−1^ (high wavenumber (HWN)).

### 3.2. Saturated fatty acids

**Figure 1.**
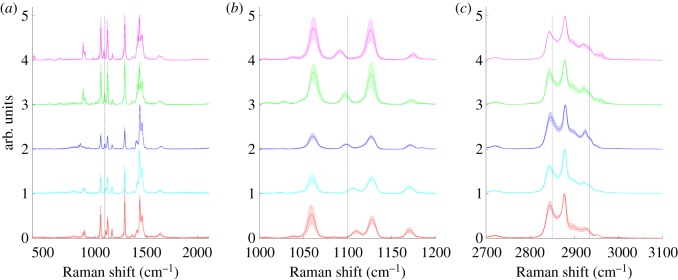
Raman spectra of five selected saturated fatty acids ranging in chain length from C14 to C22. Spectra were acquired using a 20× objective, 532 nm wavelength excitation, 10 s acquisition time and 50%/20 mW laser power, followed by smoothing, baseline subtraction and min-max scaling. Spectra are offset for clarity and each spectrum represents the mean of 3 acquisitions (solid line) with shaded standard deviation. Low wavenumber region spectra indicated that the peak position at approximately 1100 cm^−1^ (black dashed line) was sensitive to chain length (*a*). A closer view of this region of the low wavenumber spectra shows this shift more clearly (*b*). High wavenumber region spectra where the peak positions at 2850 cm^−1^ (C−H stretch CH_2_) and 2935 cm^−1^ (C−H stretch CH_3_) are highlighted with black dashed lines. (*c*) Pink: myristic acid (C14 : 0); green: palmitic acid (C16 : 0); blue: stearic acid (C18 : 0); cyan: arachidic acid (C20 : 0); red: behenic acid (C22 : 0).

**Figure 2.**
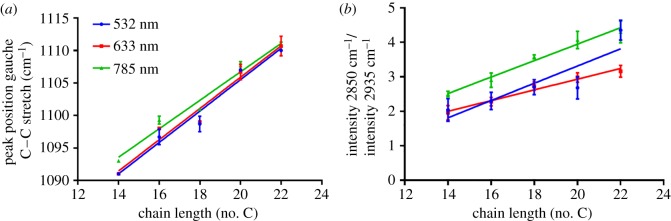
Peak position of the peak at approximately 1100 cm^−1^ corresponding to gauche C−C stretching was plotted relative to chain length of saturated fatty acids showing a linear relationship (*a*). The ratio of the peak intensity at 2850 cm^−1^ relative to peak intensity at 2935 cm^−1^ also displayed a linear correlation with chain length of saturated fatty acids (*b*). These measurements were determined using 532 nm (blue), 633 nm (red) and 785 nm (green) excitation.

Saturated FAs with a chain length from C14 to C22 were selected. Figure 1 illustrates both the LWN and HWN regions of the spectra obtained for each FA with 532 nm laser excitation (see electronic supplementary material, figures S1 and S2 for 633 nm and 785 nm laser excitation spectra, respectively). The spectra were very similar, as would be expected due to the structural similarity between compounds, only varying in number of CH_2_ groups. The primary features in the LWN region are found at approximately 1440 cm^−1^ (CH_2_ scissoring and twisting), approximately 1290 cm^−1^ (CH_3_ scissoring and twisting), approximately 1130 cm^−1^ (CH_2_ twisting) and approximately 1060 cm^−1^ (C−C stretching) [17]. The HWN region contains signals from C−H stretching. To generate a prediction method for determining chain length of an unknown saturated FA, or average chain length in a mixture of unknown saturated FAs, two characteristics were investigated. As previously observed by Czamara *et al.* [17], the position of the peak at approximately 1100 cm^−1^, corresponding to gauche C−C stretching, is dependent on chain length. Figure 2*a* demonstrates this dependence graphically, with *R*^2^ values from linear regression for all laser excitation wavelengths greater than 0.96 (electronic supplementary material, table S1). In addition, a logical structure-based hypothesis was made that the ratio of a peak associated with CH_2_ groups relative to a peak associated with CH_3_ groups would increase with increasing chain length. Peaks in the HWN region of the spectra were selected for this comparison – C−H stretching in CH_3_ groups (intensity at 2935 cm^−1^) and C−H stretching in CH_2_ groups (intensity at 2850 cm^−1^) [8,17,36]. Figure 2*b* plots the result of this investigation, with strong correlation, again giving high *R*^2^ values of 0.98 for 633 nm and 785 nm laser excitation and a poorer but still strongly correlative *R*^2^ value of 0.77 for 532 nm laser excitation (electronic supplementary material, table S1). Despite the poorer *R*^2^ value for 532 nm excitation, there was no significant difference between the best-fit line gradients from linear regression, as expected, which was also the case for the plots in figure 2*a*. Owing to the overlapping nature of peaks in Raman spectra as a result of the numerous vibrational contributions, particularly in the HWN region, it is difficult to assign peaks in this region to specific chemical groups i.e. the peak at 2935 cm^−1^ will also likely have some contribution from CH_2_ groups and that at 2850 cm^−1^ from CH_3_ groups. However, as the predominant contributor is CH_3_ groups and CH_2_ groups to 2935 cm^−1^ and 2850 cm^−1^, respectively, it is appropriate to make this approximation, and the data in figure 2*b* confirm this. Importantly, significant trends between spectral features and structural characteristics were successfully identified. Deconvolution of Raman spectra is another potential avenue to providing more species-specific information return, however, particularly in the HWN region where the spectra display one very broad Raman signal, it is incredibly difficult to fully deconvolute this into its individual component parts, which in biological samples is often millions of different species. Even in these simple pure FA species, there are broad peaks resulting from multiple overlapping bands. Therefore, the simpler ratiometric approach, which has been validated here to provide accurate information regarding chain length, could aid in simplifying analysis of multiplex samples to provide greater information return.

### 3.3. Unsaturated fatty acids

**Figure 3.**
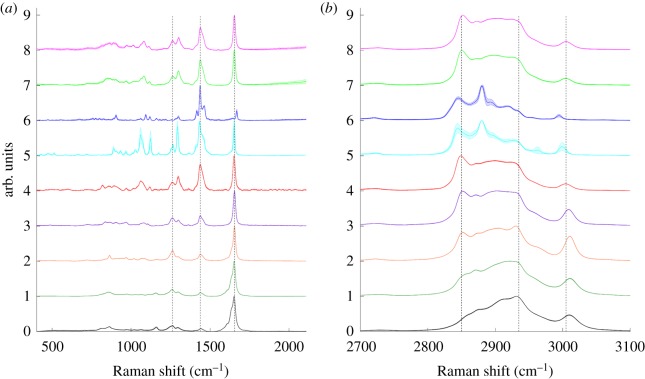
Raman spectra of eight selected unsaturated fatty acids ranging in degree of unsaturation from one C=C to four C=C. Spectra were acquired using a 20× objective, 532 nm wavelength excitation, 10 s acquisition time and 50%/20 mW laser power, followed by smoothing, baseline subtraction and min-max scaling. Spectra are offset for clarity and each spectrum represents the mean of 3 acquisitions (solid line) with shaded standard deviation. Low wavenumber region spectra where the peak positions at 1262 cm^−1^ and 1655 cm^−1^ relative to that at 1438 cm^−1^ (indicated by black dashed lines) were sensitive to degree of unsaturation (*a*). High wavenumber region spectra where the peak positions at 2850 cm^−1^ (C−H stretch CH_2_) and 2935 cm^−1^ (C−H stretch CH_3_) are highlighted with black dashed lines as well as the saturation sensitive peak at approximately 3005 cm^−1^ (*b*). Pink: palmitoleic acid (C16 : 1); green: oleic acid (C18 : 1); blue: elaidic acid (C18 : 1); cyan: petroselinic acid (C18 : 1); red: petroselinic acid melted (C18 : 1); purple: linoleic acid (C18 : 2); orange: α-linolenic acid (C18 : 3); dark green: arachidonic acid (C20 : 4); black: stearidonic acid (C18 : 4).

A series of unsaturated FAs were selected and spectra for both LWN and HWN regions were acquired (figure 3) with 532 nm laser excitation (see electronic supplementary material figures S3 and S4 for 633 nm and 785 nm laser excitation spectra, respectively). Primarily FAs with 18 carbons in the chain were selected, with varying degrees of unsaturation. Three different monounsaturated FAs (MUFAs) with 18 carbon chains were selected—oleic acid, one of the most abundant FAs found in the body; elaidic acid, the *trans* isomer of oleic acid; and petroselinic acid, an isomer of oleic acid with the C=C bond in the 6 position, as opposed to the 9 position of the FA chain. Linoleic acid (C18 : 2), α-linolenic acid (C18 : 3) and stearidonic acid (C18 : 4) were all selected as polyunsaturated FAs (PUFAs) with 18 carbon chains. In addition, the abundant C16 : 1 MUFA palmitoleic acid and C20 : 4 PUFA arachidonic acid were investigated. The most prominent spectral features for unsaturated FAs are associated with the addition of C=C double bonds. In the LWN region (figure 3*a*), this manifested with the addition of a strong peak at 1655 cm^−1^ (C=C stretch), which increased with the increasing degree of unsaturation. In addition, a similar trend was observed with the increasing relative intensity of the peak at 1262 cm^−1^ (=C−H deformation) with an increasing degree of unsaturation. In the HWN region (figure 3*b*), the presence of an additional peak at approximately 3005 cm^−1^ (=C−H stretch) indicated unsaturation, with both the position and intensity of this peak reflecting degree of unsaturation. Observation of the spectra in figure 3*a,b* showed a clear distinction between elaidic acid and petroselinic acid and the rest of the FAs. This was the result of the physical state of the FAs, with EA and PSA being the only two unsaturated FAs that were solid. The rest of the unsaturated FAs were liquid at room temperature, leading to much broader peaks. As petroselinic acid had a melting point of 33°C, it was possible to melt under hot tap water, transfer to the CaF_2_ window and acquire a spectrum before it solidified. EA had a higher melting point of 42–44°C, and re-solidified before a spectrum could be acquired. Comparing the solid and liquid spectra of PSA, it was clear that after melting, the spectral features fitted well with those of the other unsaturated FAs. For this reason, EA was omitted from trend analysis and only the spectra of PSA in liquid form were used. One important point to note about EA is that the C=C stretch peak appeared at a higher WN of 1668 cm^−1^, as opposed to approximately 1663–1665 cm^−1^ for *cis* FAs. This could have been attributed to physical state, but as the C=C stretching frequency did not change significantly for PSA upon melting, it is unlikely that this was a manifestation of physical state, and instead reflected the *trans* nature of the C=C bond present in EA, as observed previously by Czamara *et al.* [17]. Therefore, the frequency of this peak could be used as an indication of *cis*/*trans* nature; however, in biological systems *cis* is the dominating form.

**Figure 4.**
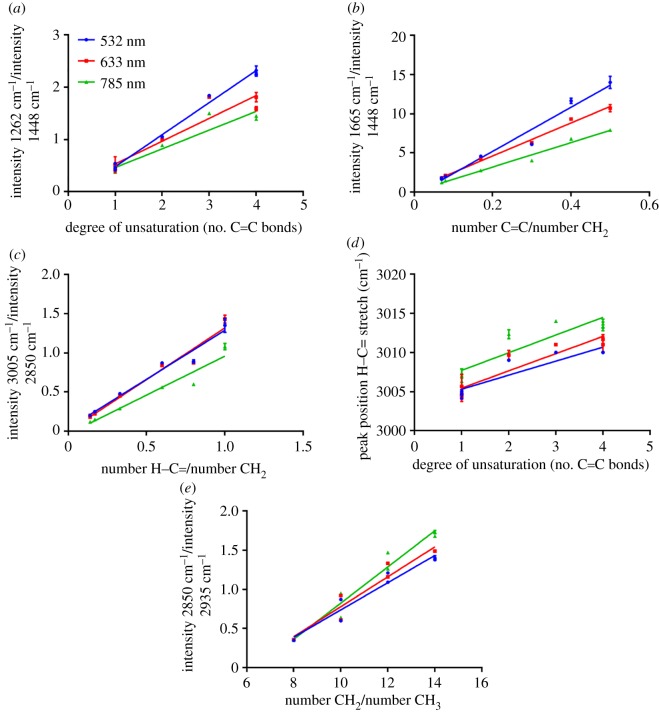
The ratio of the peak intensity at 1262 cm^−1^ relative to the intensity of the peak at 1438 cm^−1^ showed a linear correlation with number of C=C bonds (*a*). This was also the case for the ratio of peak intensity at 1655 cm^−1^ relative to the intensity of the peak at 1438 cm^−1^ when plotted against number of C=C/number of CH_2_ groups (*b*). The ratio of the peak intensity at approximately 3005 cm^−1^ relative to the intensity of the peak at 2850 cm^−1^ displayed a linear correlation with number of H−C= relative to number of CH_2_ groups for unsaturated fatty acids (*c*). Peak position of the peak at approximately 3005 cm^−1^ corresponding to H−C= stretching was plotted relative to number of C=C bonds and showed a linear relationship (*d*). The ratio of the peak intensity at 2850 cm^−1^ relative to the intensity of the peak at 2935 cm^−1^ showed a linear correlation with number of CH_2_/number of CH_3_ groups allowing chain length for unsaturated fatty acids to be determined (*e*). These measurements were determined using 532 nm (blue), 633 nm (red) and 785 nm (green) excitation.

Plots of the intensity of the peak at 1262 cm^−1^, relative to the intensity of the prominent CH_2_ twisting frequency at 1438 cm^−1^ (figure 4*a*), showed good correlation with degree of unsaturation expressed as number of C=C bonds, with R^2^ values for best-fit lines of at least 0.90 and as high as 0.99 for 532 nm excitation. Both peak intensity at 1655 cm^−1^ relative to intensity at 1438 cm^−1^ and intensity of the approximately 3005 cm^−1^ signal relative to the C−H stretching frequency of CH_2_ groups at 2850 cm^−1^, were plotted against the degree of unsaturation expressed as number of C=C bonds (electronic supplementary material, figure S3(a) and (b), respectively), showing reasonable correlation. However, better correlation was observed when plotting these ratios against the ratio of C=C to CH_2_ groups and H−C= to CH_2_ groups (table 1) in the compound (figure 4*b*,*c*, respectively). This corresponded directly to the peak ratio that was being plotted in terms of the actual groups that resulted in the signals in the spectra, and gave better linear regression fits with *R*^2^ values higher of at least 0.97 for all excitation wavelengths in figure 4*b* and at least 0.95 for all excitation wavelengths in figure 4*c* (electronic supplementary material, table S2).

Additionally, plots of the peak position of the =C−H stretch at approximately 3005 cm^−1^ signal relative to the degree of unsaturation in terms of number of C=C bonds were investigated (figure 4*d*). In this case, fits were poorer than the intensity ratio plots with *R*^2^ values of 0.85, 0.87 and 0.82 for 532 nm, 633 nm and 785 nm laser excitation, respectively (electronic supplementary material, table S2). Therefore, ratio rather than peak position-based measurements showed better performance for estimating saturation. Finally, an attempt was made to allow chain length to be predicted for unsaturated FAs. Again the intensities of the peaks at 2850 cm^−1^ (C−H stretch CH_2_) and 2935 cm^−1^ (C−H stretch CH_3_) were used. Instead of expressing chain length in terms of number of C in the FA chain, it was again important to express this as number of CH_2_ groups relative to the number of CH_3_ groups (table 1), which correlated well with the corresponding ratio (figure 4*e*), giving *R*^2^ values of at least 0.93 for all laser excitation wavelengths (electronic supplementary material, table S2). As degree of unsaturation could also be predicted for an unknown sample, the chain length in terms of number of C could be deduced from this along with CH_2_/CH_3_ ratio.

Interestingly, for the plots in figure 4*a,b* representing the intensity ratios of 1262 cm^−1^/1448 cm^−1^ and 1665 cm^−1^/1448 cm^−1^, the gradients of the best-fit lines for the three different laser excitation wavelengths were significantly different (electronic supplementary material, table S2), which was not the case for all other plots. It was expected that the gradients should be the same for all laser excitation wavelengths and, therefore, these two cases were anomalous. In both cases, the 532 nm line showed the steepest gradient, followed by the 633 nm line then the 785 nm line. This suggests that the intensity of the peaks at 1262 cm^−1^ and 1665 cm^−1^ increased relative to the peak at 1448 cm^−1^ to a greater extent upon the addition of each C=C bond, with increased laser excitation energy. One possible explanation for this observation is that there was a small resonance contribution that increased with increasing number of double bonds and therefore the 1262 cm^−1^ and 1655 cm^−1^ peaks associated with vibrations of the double bonds increased more rapidly for the higher energy excitation wavelengths.

### 3.4. Comparison of ratiometric and multivariate analysis on food oil samples

To compare the performance of the ratiometric method for determination of key structural features in a multiplex sample to a multivariate PLSR-based method, we used these approaches to characterize a selection of food oil samples. These samples were selected as they represented a relatively simple biological sample consisting primarily of mixtures of triglycerides. Triglycerides are very similar in their characteristics to free FAs as triglycerides consist of a small glycerol backbone with three FA chains attached. Czamara *et al.* [17] have already demonstrated the close similarity between the Raman spectra of the two compounds with the same FA chain. Therefore, it was assumed that by applying the ratiometric and PLSR-based prediction models to the oil spectra, the average characteristics of the triglyceride FA chains could be predicted and the performance of the two methods was compared. In addition, food oil authentication and adulteration is a major concern and therefore a rapid method for accurate assessment of oil characteristics using Raman spectroscopy could be an incredibly valuable tool in the food industry [37].

**Figure 5.**
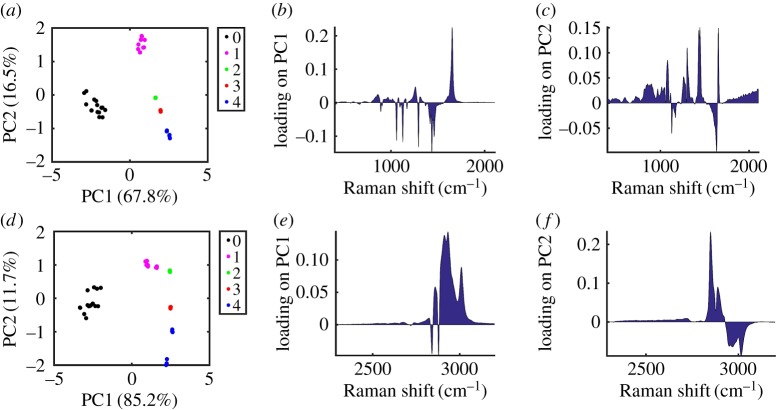
A series of Raman spectra of fatty acids (FAs) (3 replicates of each FA for low wavenumber (*a–c*) or high wavenumber (*d–f*)) were analysed using principal component analysis and results were plotted in terms of number of C=C double bonds in the fatty acid chain for principal component 1 (PC1) against principal component 2 (PC2) (*a,d*). Loadings on PC1 (*b,e*) and PC2 (*c,f*) are shown. Black: 0 C=C per FA; pink: 1 C=C per FA; green: 2 C=C per FA; red: 3 C=C per FA; blue: 4 C=C per FA.

The PLSR-based model was built by randomly splitting each dataset (3 replicates for each FA, excluding elaidic acid and solid petroselinic acid) into 70% for training and 30% for testing. Models were built using both the LWN and HWN spectral regions. PCA was also performed on each dataset and showed good separation of FA based on number of C=C bonds, with PC1 strongly separating saturated from unsaturated FAs in particular and PC2 strongly separating unsaturated FAs based on number of C=C for both LWN and HWN spectra (figure 5). As already discussed, petroselinic acid and elaidic acid did not fall in line with trends as they were solid rather than liquid at room temperature and therefore these were omitted for the purpose of this analysis; however, petroselinic acid in liquid form was included. It is probable that saturated and unsaturated FAs separated on PC1 as a result of the physical state of the FAs going from solid to liquid, respectively, leading to characteristic changes in peaks, from sharper peaks for the solid FAs, to broader peaks for the liquid state FAs, reflective of the changes expected on going from a defined crystal structure to averaging through molecular rotation in solution. From the PC1 loadings for LWN (figure 5*b*) and HWN (figure 5*e*) spectra, the peaks contributing most to this separation of saturated and unsaturated FAs were 3010 cm^−1^, 2932 cm^−1^, 2911 cm^−1^, 2858 cm^−1^, 1653 cm^−1^, 1437 cm^−1^, 1295 cm^−1^, 1127 cm^−1^ and 1061 cm^−1^. Degree of saturation, going from 1 C=C to 4 C=C separated progressively along PC2, and upon analysis of the loadings on PC2, the major contributing peaks to this separation were at 3016 cm^−1^, 2969 cm^−1^, 2951 cm^−1^, 2889 cm^−1^, and 2850 cm^−1^, 1653 cm^−1^, 1634 cm^−1^, 1445 cm^−1^, 1433 cm^−1^, 1303 cm^−1^, 1129 cm^−1^ and 1080 cm^−1^ (figure 5*c,f*). The largest contributing peaks to the PC2 loadings plots at 1653 cm^−1^, 1445 cm^−1^, 3016 cm^−1^ and 2850 cm^−1^ fall in line with the previous ratiometric observations made to correlate degree of saturation and Raman spectral signature.

**Figure 6.**
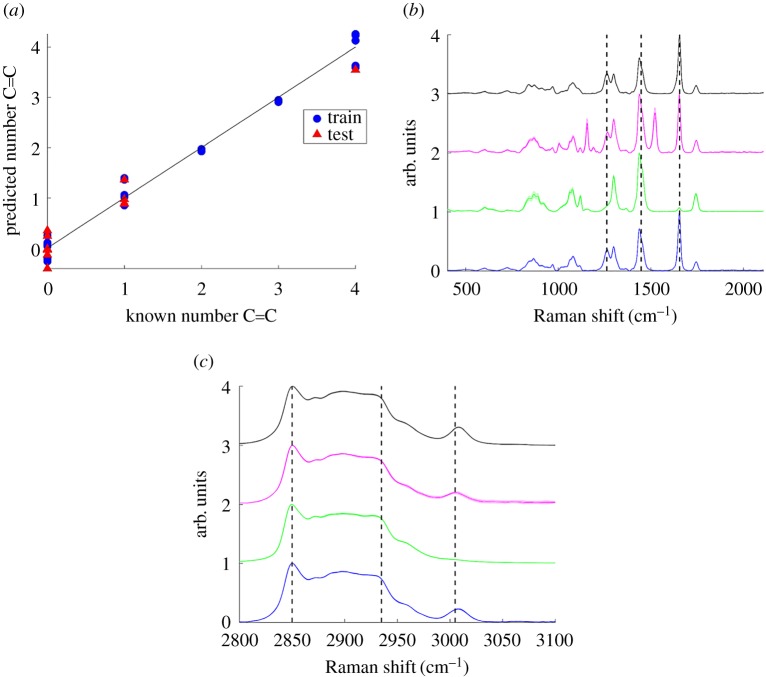
Predicted number of C=C per fatty acid versus known number of C=C per fatty acid for a partial least squares regression (PLSR) model using 2 principal components of a series of fatty acid spectra (3 replicates of each) split randomly into 70% training and 30% test data for high wavenumber spectra. MSPE was 0.042 and *R*^2^ for the training dataset was 0.98 and test dataset was 0.95. (*a*) Raman spectra of four selected commercially available food oils were measured. Three spectra of each were acquired using a 50× objective, 532 nm wavelength excitation, 10 s acquisition time and 50%/20 mW laser power (1 s for vegetable oil in (*c*)), followed by smoothing, baseline subtraction and min-max scaling. Spectra are offset for clarity and each spectrum represents the mean of 3 acquisitions (solid line) with shaded standard deviation. Low wavenumber region spectra where the peak positions at 1262 cm^−1^ and 1655 cm^−1^ relative to that at 1438 cm^−1^ (indicated by black dashed lines) were sensitive to degree of unsaturation (*b*). High wavenumber region spectra where the peak positions at 2850 cm^−1^ (C−H stretch CH_2_) and 2935 cm^−1^ (C−H stretch CH_3_) are highlighted with black dashed lines as well as the saturation sensitive peak at approximately 3005 cm^−1^ (*c*). Black: sunflower oil; pink: olive oil; green: coconut oil; blue: vegetable oil.

As PCA is an unsupervised technique, it cannot be used to predict number of C=C bonds in an unknown sample. A PLSR model was therefore investigated. PLSR was initially performed on the training datasets using 10 principal components and a plot of estimated MSPE against number of components, along with the PCA plots, revealed 2 PCs to be optimal for both LWN and HWN datasets (electronic supplementary material, figure S6). From electronic supplementary material, figure S6, either 1 or 2 PCs were selected as optimal in order to minimize MSPE without overfitting. 2 PCs were ultimately selected after consideration of the PC plots in figure 5, where PC1 only discriminated unsaturated from saturated FAs, while PC2 clearly discriminated unsaturated FAs with different degrees of unsaturation. PLSR was therefore then performed on the training datasets using 2 principal components giving a MSPE of 0.047 for the LWN spectra and 0.042 for the HWN spectra and *R*^2^ values of 0.98 for both the LWN and HWN spectra. The model was then used to predict number of C=C bonds per FA for the test datasets giving *R*^2^ values of 0.96 and 0.95 for the LWN and HWN data, respectively. Known versus predicted number of C=C bonds per FA for both the training and test data was plotted (figure 6*a*; electronic supplementary material, figure S7) and along with the MSPE and *R*^2^ values this supported PLSR as a viable model for predicting number of C=C bonds per FA. This multivariate model was applied to the food oil samples to test its performance against the ratiometric method. Both the LWN and HWN based models were tested with the HWN model giving predicted values closer to theoretical values (table 2), which was therefore used to represent PLSR-based predications.

**Table 2.** Predicted degree of saturation for food oils analysed using ratiometric and multivariate methods on Raman spectra. The average number of C=C bonds for each oil calculated using the ratiometric approach (C=C Ratio) was determined from three different peak intensity ratios 1–3. 1: 1262 cm^−1^/1438 cm^−1^; 2: 1655 cm^−1^/1438 cm^−1^; 3: 3005 cm^−1^/2850 cm^−1^. In addition, average number of C=C along with 2850 cm^−1^/2935 cm^−1^ ratio was used to predict chain length (chain length ratio). The average number of C=C bonds predicted using the PLSR model (C=C PLSR) is also given. In addition, the theoretical values for average C=C bonds per fatty acid chain (C=C theory) and C per fatty acid chain (chain length theory), taken from literature compositions [38], are given.

						C=C PLSR			
oil	1 mean ± s.e.	2 mean ± s.e.	3 mean ± s.e.	C=C ratio mean ± s.e.	chain length ratio mean ± s.e.	mean ± s.e.	mean squared prediction error	C=C theory	chain length theory
sunflower	1.45 ± 0.12	1.21 ± 0.36	1.78 ± 0.41	1.48 ± 0.19	16.9 ± 2.0	1.47 ± 0.0096	0.042	1.60	18.0
olive	0.91 ± 0.11	0.82 ± 0.33	1.29 ± 0.37	1.00 ± 0.17	16.6 ± 2.1	1.22 ± 0.077	0.042	0.93	17.8
coconut	0.42 ± 0.11	0.25 ± 0.30	0 ± 0 (no 3005 peak)	0.22 ± 0.11	14.8 ± 2.0	1.00 ± 0.0087	0.042	0.11	13.2
vegetable	1.29 ± 0.12	1.09 ± 0.35	1.44 ± 0.38	1.27 ± 0.18	17.1 ± 2.1	1.09 ± 0.020	0.042	1.30	18.0

The average Raman spectra of each of the four selected oils—sunflower, olive, coconut and vegetable—are presented in figure 6*b,c* with shaded standard deviation. It is worth noting here that, particularly with the food oil samples, and to a varying extent in the lipid standard samples, background fluorescence was observed. Where this was particularly high, it was reduced by exposing the sample to the laser over a period of time in order to bleach some of the background fluorescence. For the vegetable oil sample, the acquisition time also had to be reduced; however, it is not believed that differing background fluorescence contributions affected results, as background subtraction was performed for each spectrum to remove this and any other background contributions while avoiding over-subtraction. Each of three ratiometric predictions 1–3, corresponding to figure 4*a–c*, respectively, were used to predict the average number of C=C per FA chain in each of the oil samples (table 2). The three predictions were averaged to give an average prediction for number of C=C per FA chain. Peak position at approximately 3005 cm^−1^ (figure 4*d*) was not used for prediction due to the much poorer linear regression fits in this case. Average chain length in terms of number of C was predicted using the ratio in figure 4*e*. Each of the oil spectra was also used to create a prediction of the number of C=C per FA chain using the HWN PLSR model created (figure 6*a*). To ascertain an estimate of the expected numbers for these characteristics, a theoretical number of C=C and C per FA chain was calculated using reported FA compositions in the literature [38]. The theoretical values for average number of C=C and C per FA chain in the food oil samples were determined using FA compositions reported for sunflower oil, olive oil, coconut oil and canola oil using gas chromatography. Canola oil, a type of rapeseed oil, was used to determine theoretical values for the vegetable oil measured in this experiment as rapeseed oil was the only listed ingredient for the particular brand of vegetable oil measured. Owing to variations in the absolute composition between different brands, the theoretical values were used as an approximation of the expected values and their relation to each other for each type of food oil, with small discrepancies likely dependent on the particular brand and batch of food oil being measured. It is also possible that a small level of disparity from the theoretical values could be present as a result of the glycerol backbone groups present in the triglycerides and additional small quantities of other constituents in the oils. Despite potential discrepancies, the general trend in expected values for each oil, with respect to other oils, could be considered reliable and therefore agreement with the trend in oil saturation between each type of oil analysed, in terms of most to least saturation, was at least considered a reliable measurement for comparison.

Using the ratiometric method, the predicted average number of C=C bonds in each oil sample closely matched the theoretical value (table 2) with sunflower oil predicted at 1.48 ± 0.19 compared to the theoretical value of 1.60, olive oil predicted at 1.00 ± 0.17 compared to 0.93, coconut oil at 0.22 ± 0.11 compared to 0.11 and vegetable oil at 1.27 ± 0.18 compared to 1.30. A very strong agreement with theoretical values was observed, with theoretical values all lying within the standard error of the predicted value, and the largest discrepancy from the theoretical value being 0.12 for sunflower oil. The trend in degree of unsaturation from sunflower oil being highest, followed by vegetable oil then olive oil then coconut oil, which was determined using the ratiometric predication, followed that expected theoretically. Chain length prediction was based on a single ratiometric value (along with knowledge of the number of C=C groups) and gave a prediction of 16.9 ± 2.0 for sunflower oil compared to the theoretical value of 18.0, 16.6 ± 2.1 compared to 17.8 for olive oil, 14.8 ± 2.0 compared to 13.2 for coconut oil and 17.1 ± 2.1 compared to 18.0 for vegetable oil. These predictions were not as accurate as saturation predictions, but this could be expected as only a single ratio was used. The overall trend was good however, with the two oils with the same theoretical values of 18 having the highest predicted values of 16.9 and 17.1, a slight underestimation. Olive oil then had a theoretical chain length of 17.8 and predicted value lower than that for sunflower and vegetable oil as expected, and coconut oil had the lowest theoretical value of 13.2 and a slightly overestimated but lowest prediction of 14.8. Again all theoretical values lay within the standard errors of predicted values and the largest discrepancy was 1.6.

The PLSR model (figure 6*a*) was also used to predict number of C=C per FA based on the HWN spectra. While the model performed reasonably well, predictions in each case were further from theoretical values than the corresponding predictions using the ratiometric approach. Using the PLSR model, number of C=C per FA was predicted at 1.47 ± 0.0096 for sunflower oil, 1.22 ± 0.042 for olive oil, 1.00 ± 0.0087 for coconut oil and 1.09 ± 0.042 for vegetable oil with an MSPE of 0.042 (table 2). Predictions for all oils were further from theoretical values than for those using the ratiometric method with discrepancies from theoretical values of 0.13 (PLSR) compared to 0.12 (ratio) for sunflower oil, 0.29 (PLSR) compared to 0.07 (ratio) for olive oil, 0.89 (PLSR) compared to 0.11 (ratio) for coconut oil and 0.21 (PLSR) compared to 0.03 (ratio) for vegetable oil. In this case, the trend in degree of unsaturation determined using the PLSR model went from sunflower oil being the highest, followed by olive oil then vegetable oil then coconut oil, which did not follow that expected theoretically. In addition, the prediction using PLSR for coconut oil was particularly higher than the theoretical value. This result highlights the power and importance of simply using ratiometric analysis for predictions in certain cases as opposed to creating more complicated multivariate models that in many cases, such as this one, may not perform as well. In this case, it is shown that the ratiometric method far outperformed the PLSR model as there were a few key defining peaks in the Raman spectra that directly corresponded to bond vibrations assigned to particular chemical moieties that, as expected, correlated well with the characteristics being investigated. In the case of the PLSR model, by building a model based on the full spectra, as opposed to logical selection of a few key features, it is likely that the model was made too complex, and variations in other regions of the spectra that ultimately do not need considered to determine characteristics being investigated were overly influencing the ultimate prediction. The ratiometric approach also allowed for chain length to be easily predicted using an additional ratio, which would not be possible simply using a single PLSR-based model.

## 4. Conclusion

This study has demonstrated powerful evidence for the use of ratiometric analysis for dissecting complicated spectral information in order to extract key features of biological samples. Both degree of unsaturation and chain length of FA species could be predicted, allowing the identity of individual lipid species to be inferred. While multivariate analysis using PLSR was able to predict degree of unsaturation reasonably well in multiplex food oil samples and could be a useful tool in certain cases, predictions using the ratiometric approach were much closer to the theoretical degree of unsaturation. In addition to predicting degree of unsaturation in a selection of food oil samples to a high degree of accuracy, with all theoretical values lying within the standard error of the prediction and the largest discrepancy from the theoretical value being 0.11, the ratiometric approach also allowed chain length to be predicted with good accuracy, supporting the power of this ratiometric approach. This study outlines a strong foundation for a bottom-up and chemical knowledge-based approach to understanding the detailed composition of biological samples represented in Raman spectra by consideration of the origin of spectral signals in terms of chemical groups. By careful analysis of Raman spectra of FAs, and relating signals back to the chemical bonds that they originated from, this study successfully demonstrated the power of simple ratiometric analysis to enhance understanding of complicated biological systems using Raman spectroscopy. In addition, the ratiometric approach was applied to a simple example of a multiplex system – food oils. This is a particularly significant application that would allow rapid assessment of food oils for authentication and adulteration, a highly topical problem. While there may be better approaches using Raman spectroscopy and chemometrics to identify adulterated foodstuffs, this served as a topical and relevant application for demonstration of the utility of this ratiometric approach, and provided an alternative approach to this problem. This comprehensive study allows a foundation for a ratiometric-based approach to analysing multiplexed biological samples to be built, starting from a single class of lipid species and identifying key progressive trends in Raman peak ratios correlated to structural features of the selected species. This strong foundation is necessary for building ratiometric-based models to allow higher species-specific information return currently not possible from Raman analysis to be gained.

## Supplementary Material

Electronic supplementary information

## Data accessibility

Raw data and MATLAB scripts available at http://dx.doi.org/10.15129/f3f92bdb-5b8e-4f4e-96b0-b377a9d1ea5e [39].
