# Supplementary material for: Ratiometric analysis using Raman spectroscopy as a powerful predictor of structural properties of fatty acids
Source: R Soc Open Sci. 2018 Dec 12;5(12):181483. doi: 10.1098/rsos.181483 (PMC6304136; doi:10.1098/rsos.181483)
Supplement: Electronic supplementary information [file rsos181483supp1.pdf]

Electronic Supplementary Information

Ratiometric analysis using Raman spectroscopy as a powerful predictor of structural properties of fatty acids

Lauren E. Jamieson,[a] Angela Li, [a†], Karen Faulds[a] and Duncan Graham [*a]

[a]Centre for Molecular Nanometrology, WestCHEM, Department of Pure and Applied Chemistry, Technology and Innovation Centre, University of Strathclyde, 99 George Street, Glasgow, G1 1RD, UK.

*duncan.graham@strath.ac.uk

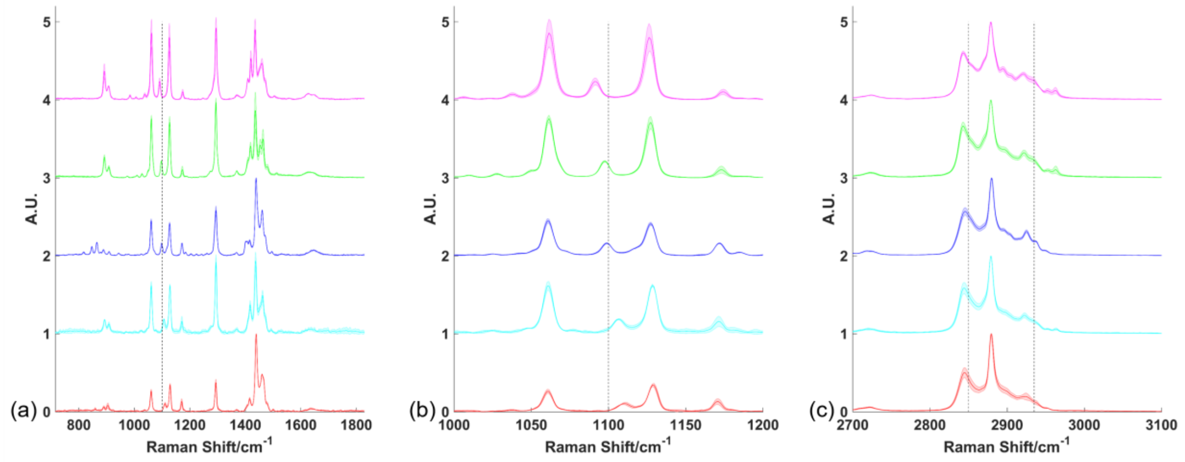

Figure S1 Raman spectra of five selected saturated fatty acids ranging in chain length from C14 to C22. Spectra were acquired using a 20× objective, 633 nm wavelength excitation, 10 s acquisition time and 50%/10 mW laser power, followed by smoothing, baseline subtraction and min-max scaling. Spectra are offset for clarity and each spectrum represents the mean of 3 acquisitions (solid line) with shaded standard deviation. Low wavenumber region spectra indicated that the peak position at ~1100 cm^{-1} (indicated by black dashed line) was sensitive to chain length (a). A closer view of this region of the low wavenumber spectra shows this shift more clearly (b). High wavenumber region spectra where the peak positions at 2850 cm^{-1} (C–H stretch CH_2) and 2935 cm^{-1} (C–H stretch CH_3) are highlighted with black dashed lines (c). Pink: myristic acid (C14:0); green: palmitic acid (C16:0); blue: stearic acid (C18:0); cyan: arachidic acid (C20:0); red: behenic acid (C22:0).

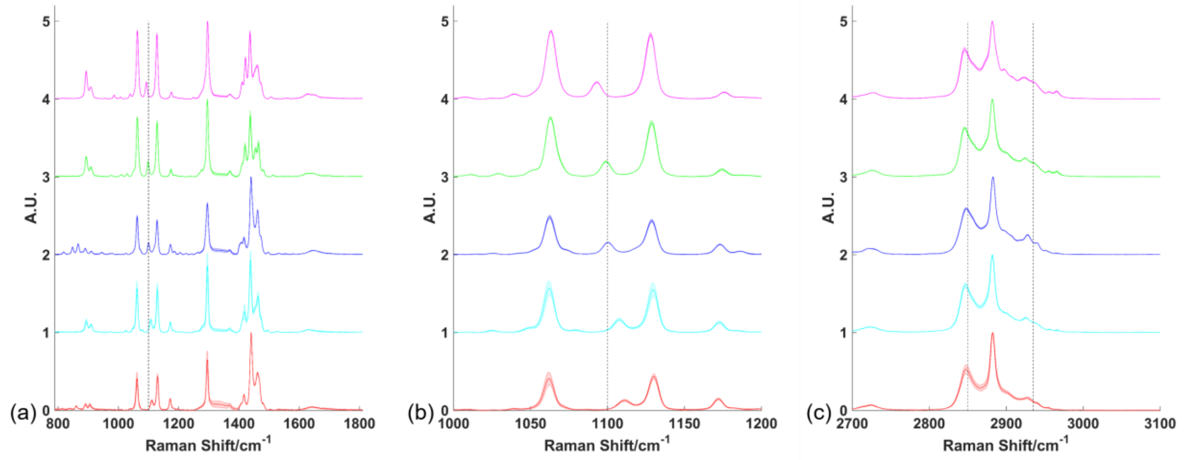

Figure S2 Raman spectra of five selected saturated fatty acids ranging in chain length from C14 to C22. Spectra were acquired using a 20× objective, 785 nm wavelength excitation, 10 s acquisition time and 50%/95 mW laser power, followed by smoothing, baseline subtraction and min-max scaling. Spectra are offset for clarity and each spectrum represents the mean of 3 acquisitions (solid line) with shaded standard deviation. Low wavenumber region spectra indicated that the peak position at ~1100 cm^{-1} (indicated by black dashed line) was sensitive to chain length (a). A closer view of this region of the low wavenumber spectra shows this shift more clearly (b). High wavenumber region spectra where the peak positions at 2850 cm^{-1} (C–H stretch CH_2) and 2935 cm^{-1} (C–H stretch CH_3) are highlighted with black dashed lines (c). Pink: myristic acid (C14:0); green: palmitic acid (C16:0); blue: stearic acid (C18:0); cyan: arachidic acid (C20:0); red: behenic acid (C22:0).

Table S1 Straight line fit parameters for linear regression on the plots in Figure 2, Figure S1 and Figure S2 along with R^2 values for each plot.

Plot	Laser wavelength (nm)	Gradient (m) ± SE	y-intercept (c) ± SE	R^2	Gradients significantly different?
**Peak position gauche C–C stretch (Figure 2(a))**	532	2.415 ± 0.2412	1057 ± 4.394	0.9710	No
	633	2.405 ± 0.2612	1058 ± 4.759	0.9658	
	785	2.19 ± 0.2051	1063 ± 3.737	0.9744	
**2850 cm⁻¹/2935 cm⁻¹ (Figure 2(b))**	532	0.2500 ± 0.07924	−1.686 ± 1.444	0.7684	No
	633	0.1550 ± 0.01399	−0.168 ± 0.2549	0.9761	
	785	0.2385 ± 0.02072	−0.823 ± 0.3775	0.9779	

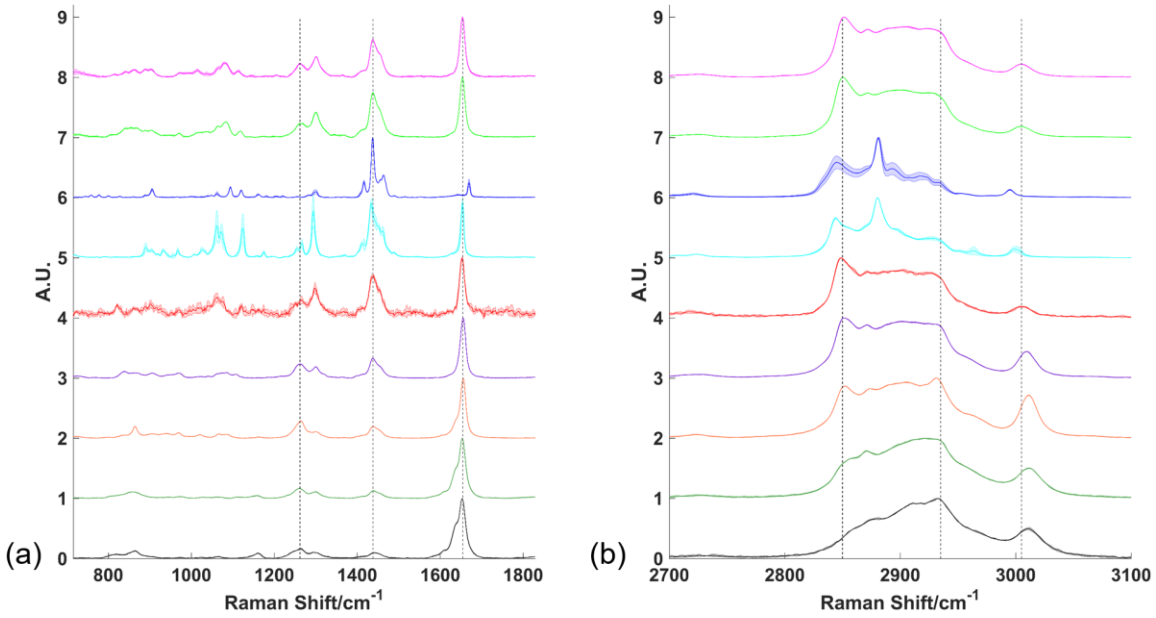

Figure S3 Raman spectra of eight selected unsaturated fatty acids ranging in degree of unsaturation from one C=C to four C=C. Spectra were acquired using a 20× objective, 633 nm wavelength excitation, 10 s acquisition time and 50%/10 mW laser power, followed by smoothing, baseline subtraction and min-max scaling. Spectra are offset for clarity and each spectrum represents the mean of 3 acquisitions (solid line) with shaded standard deviation. Low wavenumber region spectra where the peak positions at 1262 cm^{-1} and 1655 cm^{-1} relative to that at 1438 cm^{-1} (indicated by black dashed lines) were sensitive to degree of unsaturation (a). High wavenumber region spectra where the peak positions at 2850 cm^{-1} (C–H stretch CH_2) and 2935 cm^{-1} (C–H stretch CH_3) are highlighted with black dashed lines as well as the saturation sensitive peak at ~3005 cm^{-1} (b). Pink: palmitoleic acid (C16:1); green: oleic acid (C18:1); blue: elaidic acid (C18:1); cyan: petroselinic acid (C18:1); red: petroselinic acid melted (C18:1); purple: linoleic acid (C18:2); orange: α-linolenic acid (C18:3); dark green: arachidonic acid (C20:4); black: stearidonic acid (C18:4).

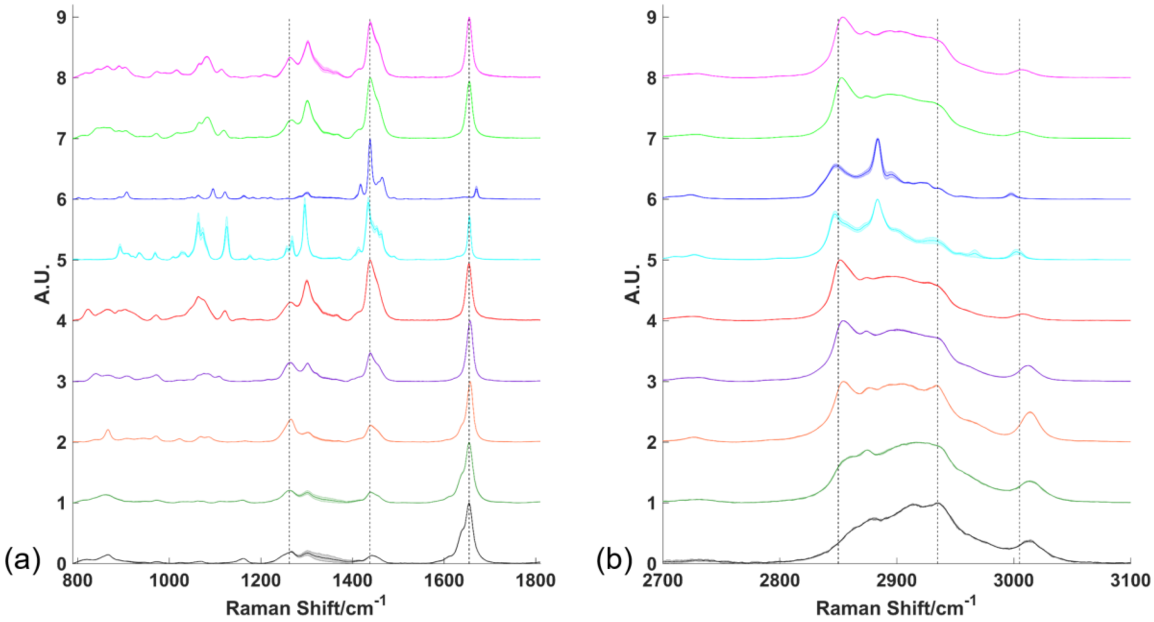

Figure S4 Raman spectra of eight selected unsaturated fatty acids ranging in degree of unsaturation from one C=C to four C=C. Spectra were acquired using a 20× objective, 785 nm wavelength excitation, 10 s acquisition time and 50%/95 mW laser power, followed by smoothing, baseline subtraction and min-max scaling. Spectra are offset for clarity and each spectrum represents the mean of 3 acquisitions (solid line) with shaded standard deviation. Low wavenumber region spectra where the peak positions at 1262 cm^{-1} and 1655 cm^{-1} relative to that at 1438 cm^{-1} (indicated by black dashed lines) were sensitive to degree of unsaturation (a). High wavenumber region spectra where the peak positions at 2850 cm^{-1} (C−H stretch CH_2) and 2935 cm^{-1} (C−H stretch CH_3) are highlighted with black dashed lines as well as the saturation sensitive peak at ~3005 cm^{-1} (b). Pink: palmitoleic acid (C16:1); green: oleic acid (C18:1); blue: elaidic acid (C18:1); cyan: petroselinic acid (C18:1); red: petroselinic acid melted (C18:1); purple: linoleic acid (C18:2); orange: α-linolenic acid (C18:3); dark green: arachidonic acid (C20:4); black: stearidonic acid (C18:4).

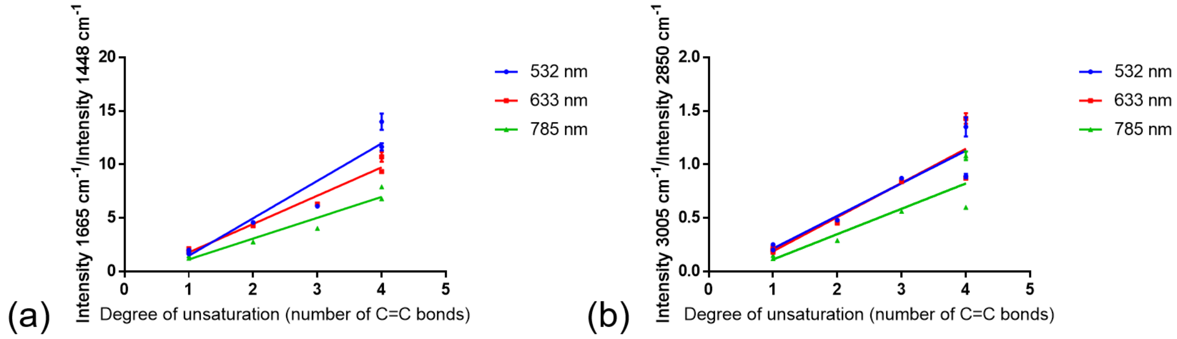

Figure S5 The ratio of the peak intensity at 1665 cm^{-1} relative to the intensity of the peak at 1448 cm^{-1} (a) and the ratio of the peak intensity at ~3005 cm^{-1} relative to the intensity of the peak at 2850 cm^{-1} (b) showed poorer linear regression fits when plotted against the number of C=C bonds instead of the ratio of C=C to CH_2 groups (Figure 4(b)) and H-C= to CH_2 groups (Figure 4(c)) respectively. R^2 values of 0.93 using 532 nm excitation (blue), 0.98 using 633 nm excitation (red) and 0.95 using 785 nm excitation (green) were obtained for the plots in (a) while R^2 values of 0.91 using 532 nm excitation (blue), 0.88 using 633 nm excitation (red) and 0.84 using 785 nm excitation (green) were obtained for the plots in (b).

Table S2 Straight line fit parameters for linear regression on the plots in Figure 4, Figure S3 and Figure S4, along with R^2 values for each plot.

Plot	Laser wavelength (nm)	Gradient (m)	y-intercept (c)	R^2	Gradients significantly different?
**1262 cm^{-1}/1438 cm^{-1} (Figure 4(a))**	532	0.613 ± 0.02426	−0.134 ± 0.06354	0.9922	Yes
	633	0.4361 ± 0.06521	0.096 ± 0.1708	0.8995	
	785	0.358 ± 0.05141	0.106 ± 0.1346	0.9065	
**1655 cm^{-1}/1438cm^{-1} (Figure 4(b))**	532	28.15 ± 2.244	−0.4347 ± 0.6263	0.9692	Yes
	633	21.06 ± 0.779	0.3968 ± 0.2174	0.9932	
	785	15.59 ± 0.9261	0.07958 ± 0.2584	0.9827	
**3005 cm^{-1}/2850 cm^{-1} (Figure 4(c))**	532	1.249 ± 0.0959	0.03831 ± 0.05348	0.9714	No
	633	1.319 ± 0.1162	−0.0004112 ± 0.06482	0.9626	
	785	0.9933 ± 0.1056	−0.03551 ± 0.0589	0.9465	
**Peak position H−C= stretch (Figure 4(d))**	532	1.787 ± 0.3419	3004 ± 0.8954	0.8452	No
	633	2.196 ± 0.387	3003 ± 1.014	0.8655	
	785	2.255 ± 0.4754	3005 ± 1.245	0.8182	
**2850 cm^{-1}/ 2933 cm^{-1} (Figure 4(e))**	532	0.1738 ± 0.01968	−1 ± 0.2285	0.9397	No
	633	0.1907 ± 0.02378	−1.128 ± 0.2761	0.9279	
	785	0.2308 ± 0.02452	−1.483 ± 0.2848	0.9466	

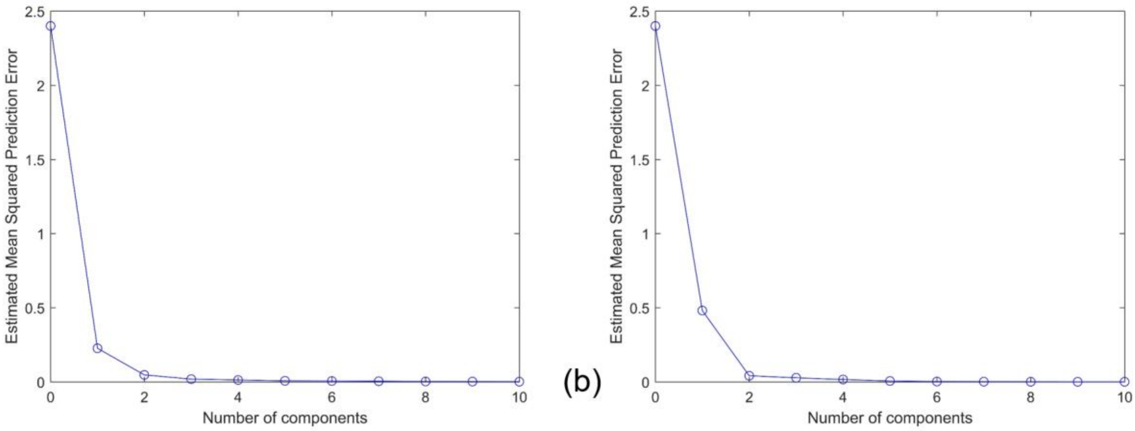

Figure S6 Estimated mean squared prediction error plotted against number of components for partial least squares regression analysis performed on a training set (70%) of a series of fatty acid spectra (3 replicates of each) for prediction of number of C=C bonds per fatty acid using low wavenumber spectra (a) and high wavenumber spectra (b).

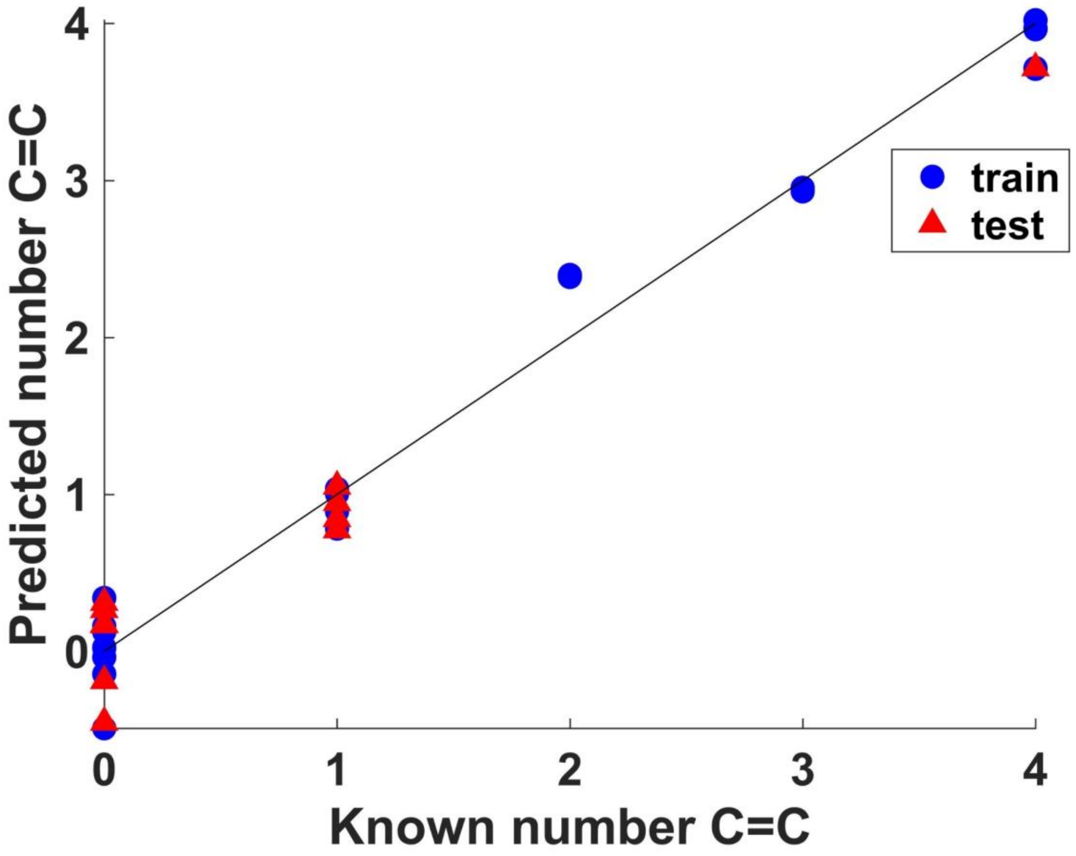

Figure S7 Predicted number of C=C per fatty acid vs. known number of C=C per fatty acid for a partial least squares regression (PLSR) model using 2 principal components of a series of fatty acid spectra (3 replicates of each) split randomly into 70% training and 30% test data for low wavenumber spectra. Mean squared prediction error was 0.47 and R^2 for the training dataset was 0.98 and test dataset was 0.96.
